# Motility-related protein-1 (MRP-1/CD9) expression can predict disease-free survival in patients with squamous cell carcinoma of the head and neck

**DOI:** 10.1038/sj.bjc.6601542

**Published:** 2004-01-20

**Authors:** P Mhawech, P Dulguerov, E Tschanz, C Verdan, C Ares, A S Allal

**Affiliations:** 1 Department of Pathology, Geneva University Hospital, CMU, Michel-Servet, 1, Geneva 1211, 4, Switzerland; 2 Division of Head and Neck Surgery, Geneva University Hospital, CMU, Michel-Servet, 1, Geneva 1211, 4, Switzerland; 3 Division of Radiation Oncology, Geneva University Hospital, CMU, Michel-Servet, 1, Geneva 1211, 4, Switzerland

**Keywords:** CD9 expression, IHC, SCC of head and neck, DFS

## Abstract

CD9 is a transmembrane protein that has been implicated in cell adhesion, motility and proliferation, and numerous studies have demonstrated the prognostic value of its expression in different solid tumours. The purpose of this study is to determine the predictive value of CD9 in squamous cell carcinoma (SCC) of the head and neck. A total of 153 cases were examined for CD9 expression using immunohistochemistry applied on formalin-fixed, paraffin-embedded tissue. Cases were stratified in two categories depending on CD9 expression, as positive (⩾50% positive cells) or reduced (<50%). In all, 108 cases were positive for CD9 (85 cases with membranous, and 23 with both membranous and cytoplasmic staining) and 45 reduced expression. Reduced CD9 expression was significantly associated with high grade (*P*=0.0007) and lower disease-free survival (DFS) (*P*=0.017). The latter retained its significance in the multivariate analysis. When the 23 cases with both membranous and cytoplasmic patterns were studied as a separate subgroup, there were significant associations between CD9 expression and tumour grade (*P*=0.025) (95% CI 11–68), tumour stage (*P*=0.08) (95% CI 3.5–86) and the occurrence of any failure (*P*=0.083) (95% CI −1.7–57). Immunohistochemical CD9 expression proved to be an independent prognostic factor in SCC of the head and neck, and it may detect patients at a high risk of recurrence. In addition, the cytoplasmic pattern seems to have an even more significant value. However, this finding is limited to the small number of cases with this pattern.

Despite the important advances made in the management of squamous cell carcinoma (SCC) of the head and neck, patients still have poor prognosis with a 5-year disease-free survival (DFS) of about 50% (Landis *et al*, 1999). The prognostic factors are mostly confined to the histopathologic and clinical parameters such as grade, stage, pattern of invasion, location and lymph node metastasis (Crissman *et al*, 1984; Pera *et al*, 1986; Wiernik *et al*, 1991; DeStefani *et al*, 1997). Recently, numerous molecular markers such as cell cycle regulators, cell adhesion proteins, oncogenes and tumour-suppressor genes have been investigated in SCC of the head and neck, and some of these markers have shown promising results for future use (Schoelch *et al*, 1999; Grabenbauer *et al*, 2000; Pukkila *et al*, 2001; Khademi *et al*, 2002; Khan *et al*, 2002; Liu *et al*, 2003).

Disruption of cell adhesion and alteration of cell motility play an important role in cancer cell invasion and metastasis. Previously, CD9 was reported to be of prognostic significance in adenocarcinoma of the lung, colon, breast, pancreas and SCC of the oesophagus and the oral cavity. Motility-related protein (MRP-1)/CD9 is a member of the transmembrane 4 superfamily (TM4SF) which is involved in cell growth, adhesion and motility. Of the 20 known members, five – MRP1/CD9, ME491/CD63, KAI1/CD82, CD151, CD81 – may be implicated in cell migration, proliferation and tumour cell metastasis (Miyake *et al*, 1991; Ikeyama *et al*, 1993). The motility-related protein/CD9 is so far the best characterised of the TM4SF members. It is located on chromosome 12 (12p13) and is widely distributed among all cell types. CD9 is capable of interacting with other transmembrane proteins such as integrins and other tetraspanins to form a complex, which facilitates cell adhesion, motility and signalling (Hemler *et al*, 1996). Thus, CD9 appears to have an important role in inhibiting cell motility in numerous neoplastic cell lines (Miyake *et al*, 1991).

Studies evaluating the expression of this CD9 protein in SCC of the head and neck are nonexistent, save only for one study, and it was limited to the SCC of the oral cavity (Kusukawa *et al*, 2001). Thus, the aim of this study is to assess the utility of the CD9 protein expression in predicting DFS of patients with SCC arising in the head and neck after treatment with radiotherapy (RT) with or without chemotherapy.

## MATERIALS AND METHODS

### Patients population

A retrospective study covering 7 years (1992–1999) was conducted. The criteria for inclusion were patients with no prior treatment, histologic diagnosis performed in our department and adequate material for analysis. Tumours with a nasopharyngeal origin were excluded from the study. From 201 registered patients, 153 met our criteria for inclusion. The patient and tumour characteristics are summarised in Table 1

**Table 1:** Patients and tumours characteristics

Median age (years) (range)	59 (35–90)
Sex M : F	122/31
*Tumour sublocation*	
Oral cavity	8
Oropharynx	93
Hypopharynx	25
Larynx	27
*TN classification and stages (UICC 1997)*	
T1–2	64
T3–4	89
NO	72
N+	81
Stage I–II	35
Stage III–IV	118
Histology grading	
Well differentiated (G1)	64
Moderately differentiated (G2)	66
Poorly differentiated (G3)	23

. After the initial diagnosis, patients were treated with radical RT with or without chemotherapy. All patients were regularly followed up by the otolaryngologist, and the radio-oncologist. Therapy modality and follow-up data were retrieved from the registry at the Division of Radiation Oncology. The median follow-up for the surviving patients was 65 months (range 14–123).

### Treatment

All patients received the same accelerated RT schedule using concomitant boost technique. The latter has been described previously in detail (Allal *et al*, 1999). The planned total dose was 69.9 Gy, delivered in 41 fractions over a period of 38 days. The basic course was given to a total dose of 50.4 Gy over 5.5 weeks. The boost to the initial sites of macroscopic tumour involvement consisted of 19.5 Gy, and was given as a second daily fraction, starting the last day of the second week of the basic treatment. According to our institutional policy, 20 patients (13%) underwent a planned neck dissection prior to RT, either radical or modified radical, while one patient had simple excisions of lymph node metastases. Otherwise, surgery was reserved for salvage of locoregional failures.

Chemotherapy was given to 38 patients (25%), usually for patients presenting with T3–4 or N2–3 tumours, if their medical condition was judged fit enough to tolerate multimodality treatment. Except for two patients who received induction chemotherapy, all patients received one or more cycles of cisplatin and 5-FU-based chemotherapy, concomitantly with RT.

### Immunohistochemistry

**Figure 1:**
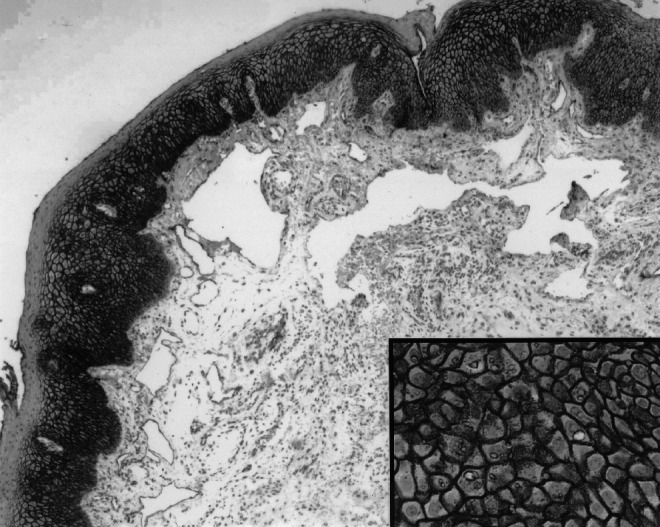
Tissue from normal laryngeal mucosae showed positive staining for CD9 using IHC in the squamous epithelium, while the connective tissue is negative. The squamous epithelial cells are positive with membranous pattern (inset).

The tissue analysed consisted of initial (pretreatment) biopsies. The original diagnosis was reviewed by two pathologists (PM, ET) and the histologic grade was assessed using the WHO system. Immunohistochemistry (IHC) was performed on paraffin-embedded tissues. Tissue sections were deparaffinised with xylene and washed with ethanol. For immunostaining enhancement, pretreatment by microwave oven in 0.01 M citrate buffer (pH 6.0) at 98°C for 30 min was done. Sections were incubated with a monoclonal anti-CD9 antibody (Novacastra, Newcastle, UK), diluted at 1 : 20. Endogenous peroxidase was blocked with 0.3% hydrogen peroxidase for 5 min. Sections were then incubated with mouse Envision horseradish peroxidase (HRP) for 30 min. IHC was performed using the automated stainer Dako ‘Autostainer’ (Dako, Copenhagen, Denmark). These incubations were performed at room temperature, and sections were washed by Tris buffer saline between incubations. Diaminobenzidine complex was used as chromogen. Tissue sections from cases of fibroadenoma were taken as positive controls. Staining of normal squamous epithelium with anti-CD9 showed strong membranous positivity (Figure 1

). In negative controls, a normal goat serum was used in place of the primary antibody, resulting in a lack of detectable staining. Evaluation of the IHC was done twice by one pathologist (PM) at 2 weeks interval. All the tissue on the slide was scanned for CD9 expression, and the average was calculated for the entire slide. Samples for CD9 expression were stratified into two categories, depending on the percentage of CD9-positive cells of any pattern (cytoplsamic/membranous). Cases showing ⩾50% CD9-positive tumour cells were classified as ‘positive’, while cases with <50% stained tumour cells were considered as ‘reduced’ cases.

### Statistical analysis

The Mann–Whitney and Kruskal–Wallis tests were used to compare the CD9 median values of the different subgroups. The actuarial overall and DFS rates were calculated using the Kaplan–Meier method. For comparison between curves, the log-rank test was used. Multivariate analyses based on Cox proportional hazards standard model were used to identify the most significant factors related to outcomes. *P*-values of 0.05 or less were considered significant. All analysis was performed with the StatView V 5.0.1 software.

## RESULTS

### Overall results

At last follow-up, 58 patients were alive, and 95 had died. In all, 56 patients presented with one or more events. A total of 33 patients presented with persistent or recurrent local disease, 15 with regional disease (five regional only) and 19 with distant metastases (16 distant only). At 5 years, actuarial DFS was 58% (95% CI 0.49–0.67) and overall survival was 38% (95% CI 0.30–0.46).

### CD9 expression and clinicopathologic associations

**Figure 2:**
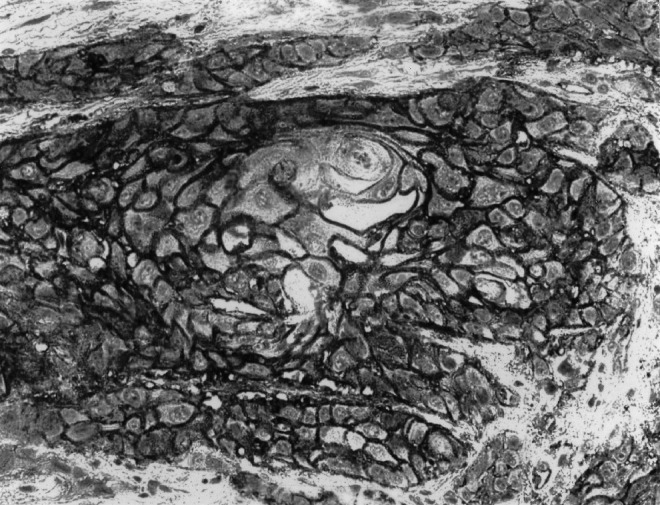
An example of positive staining for CD9, where 90% of tumour cells showed a strong reactivity with a membranous pattern.

On CD9 evaluation, 108 cases were CD9 positive with any staining pattern (cytoplasmic/membranous) and 45 showed reduced expression. Of the 108 positive cases, 85 had membranous staining and 23 had both membranous and cytoplasmic staining (Figures 2

, 3

**Figure 3:**
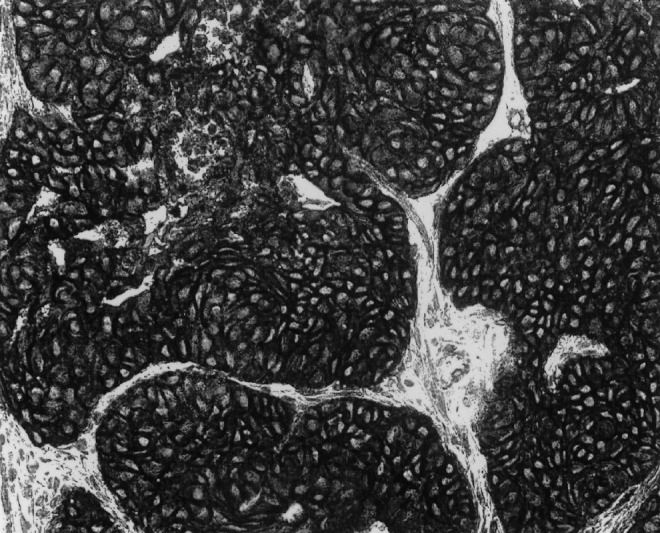
An example of the combined pattern where >90% of tumour cells showed CD9 expression in both membranous and cytoplasmic patterns.

). CD9 are expressed in 100, 70 and 10% of G1, G2 and G3 cases, respectively. When the CD9 expression was compared with various clinical features, there was no significant association between the CD9 expression and T stage (*P*=0.41), lymph node status (*P*=0.53), UICC stage grouping system (0.77) or tumour sublocation (*P*=0.3). However, there was a highly significant association between CD9 expression and tumour grade (*P*=0.0007).

### Univariate and multivariate analyses

**Figure 4:**
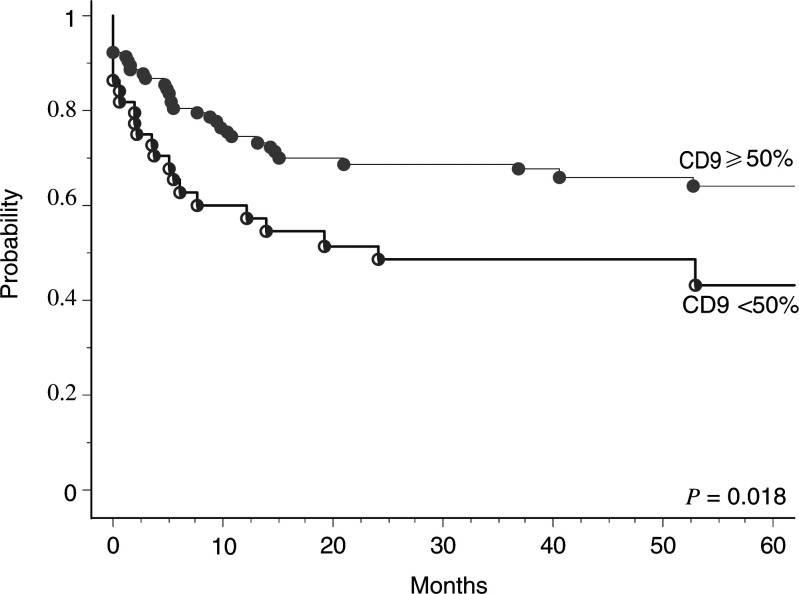
Actuarial DFS after therapy according to CD9 expression (all staining patterns (log-rank test of 0.018)).

**Figure 5:**
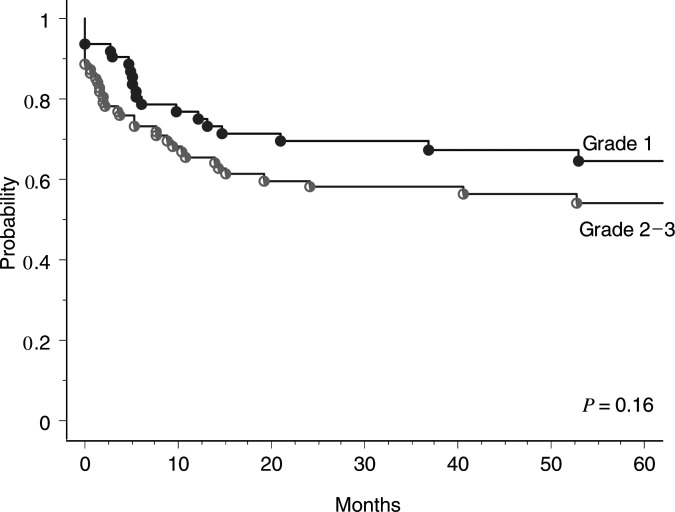
Actuarial DFS according to the histological grading (G1 *vs* G2–3) (log-rank test 0.16).

In the univariate analysis, besides advanced T (*P*=0.013) and N categories (*P*=0.002) and UICC stage (*P*=0.002), the reduced expression of CD9 was significantly associated with lower 5-year DFS (43% *vs* 64%, *P*=0.018) (Figure 4

). Histology grading (G1 *vs* G2–3) (Figure 5

) and treatment category (with or without chemotherapy) did not correlate significantly with DFS.

Factors significantly influencing DFS in univariate analysis were included in the Cox model (except linked variables). In such a model, T-category (*P*=0.024), N-category (*P*=0.012) and CD9 expression (*P*=0.017) retained their significance for DFS, and can consequently be considered as independent factors in predicting tumour failure. The relative risks associated with these factors are listed in Table 2

**Table 2:** Cox proportional hazards model for DFS

**Variables**	**RR**	**95% Cl**	***P*-value**
T -category: T1–2/T3–4	0.51	0.29–0.91	0.024
N -category: N0/N1–3	0.48	0.27–0.85	0.012
CD9 expression: <50%/⩾50%	1.9	1.12–3.33	0.017

DFS=disease-free survival; RR=relative risk; CI=confidence interval.

. In a similar model, when substituting CD9 expression by tumour grading (G1 *vs* G2–3), the impact of the latter on DFS remained nonsignificant (*P*=0.3).

### Subgroup analysis

By contrast to tumours expressing only membranous CD9, the 23 tumours with both membranous and cytoplasmic staining showed a trend toward significant association with tumour stage (*P*=0.08) (95% CI 3.5–86) and disease failure (*P*=0.083) (95% CI −1.7–57), while the association with tumour grade (G1 *vs* G2–3) was less strong (*P*=0.025 *vs* 0.0006) (95% CIs 11–68 *vs* 10–37). Thus, in this subgroup (membranous and cytoplasmic staining), the mean tumour CD9 values in patients with and without any oncological event were 37 and 65%, respectively (*P*=0.08). The impact of CD9 expression on the 5-year actuarial DFS was more significant in the subgroup with both membranous and cytoplasmic pattern compared to the subgroup with membranous pattern only (*P*=0.009 *vs* 0.04).

## DISCUSSION

The present study of 153 patients with a long-term follow-up is the first to investigate the value of CD9 protein in SCC of the head and neck. Among all parameters analysed, such as tumour grade, location, age, lymph node status, UICC stage classification, tumour grade was the only parameter to show a significant association with CD9 expression: low grade tumours appeared to express the CD9 protein more frequently. On the other hand, the present study indicates that reduced CD9 expression by tumour cells seems to predict disease progression after RT with or without chemotherapy. The link between reduced CD9 expression and tumour failure was independent of tumour T and N –categories, as found in the multivariate analysis. If CD9 is to be considered for clinical used in the future, that might add an additional 18 pound sterling to the patient's bill (which is an almost insignificant cost).

The expression of CD9 using the IHC technique has been identified in numerous solid tumours. By evaluating CD9 expression in oesophageal SCC, Uchida *et al* found that the 5-year survival rate of patients with reduced CD9 is worser than those with positive CD9 expression (Uchida *et al*, 1999). Miyake *et al* (1996) found that reduced CD9 expression is strongly associated with a high frequency of metastatic lymph node in breast cancer patients, and might be used to identify patients at high risk for disease recurrence. Huang *et al* (1998) also showed the prognostic value of CD9 in patients with breast cancer. In their study, CD9-positive expression predicted a better rate of DFS. Higashiyama *et al* (1997) showed an inverse relation between CD9 expression and DFS in patients with lung adenocarcinoma. In all these studies, CD9 expression was seen as a membranous pattern. However, there are two investigations in the literature describing both the cytoplasmic and membranous patterns, as noted in our study in addition to the usual membranous staining. The first was by Kawashima *et al* (2002) and the second was by Houle *et al* (2002)). In the former, CD9 expression was evaluated in different types of brain tumours, and they concluded that CD9 expression in astrocytic tumours correlated with their malignancy and thus CD9 protein may have a different role in brain tumours than solid tumours elsewhere. However, the cytoplasmic staining was not commented on. The latter evaluated CD9 expression in ovarian carcinoma. Loss of CD9 was found to be associated with tumour grade and, in particular, cytoplasmic CD9 expression with higher tumour grade. However, the study consisted of a small number of cases (38 cases), and the cytoplasmic staining was seen in a very few cases and very focally. Thus, the authors themselves did not reach any conclusion on the significance of their finding due to the size of the sample analysed. In addition, the cutoff value for positivity was not mentioned. In our study, we find that the impact of CD9 expression on DFS was more significant in the subgroup with both membranous and cytoplasmic patterns, compared to the subgroup with membranous pattern only. In addition, besides its association to tumour grade, there was an association with another prognostic parameter, tumour stage. However, due to the small sample size, we are not able to assess the true significance of this finding and we cannot really draw an accurate conclusion. Studies with larger samples are needed to clarify the true meaning of this finding. The only study with results directly comparable to ours is that by Kusukawa *et al*, who found a strong association between loss of CD9 expression and high incidence of lymph node metastasis and poor prognosis of patients with SCC of the oral cavity. However, in this study, the cutoff value for positive tumour was not clearly stated, and multivariate study using the Cox regression analysis was not done.

The ability of neoplastic cells to invade the surrounding tissue and metastasis to distant organs comes from the disruption of cell adhesion and alteration of cell motility. CD9, which plays an important role in cell growth, cell adhesion and motility, seems to be an ideal candidate. In this study, we showed that CD9 expression in SCC of the head and neck may be an independent factor in predicting DFS, and consequently it may have a potential use in identifying patients with higher risk of recurrence after therapy.
